# Supplementary material for: Identification of photoperiod-induced specific miRNAs in the adrenal glands of Sunite sheep (Ovis aries)
Source: Front Vet Sci. 2022 Jul 22;9:888207. doi: 10.3389/fvets.2022.888207 (PMC9354845; doi:10.3389/fvets.2022.888207)
Supplement: Supplementary file 1 [file Data_Sheet_1.ZIP › Supplementary file/miRDeep2 file.zip!/miRDeep2 file/Know_Structure/SP42A3/oar-let-7f.pdf]

miRBase precursor : oar-let-7f
Total read count : 698189
oar-let-7f read count : 698064
remaining reads : 125

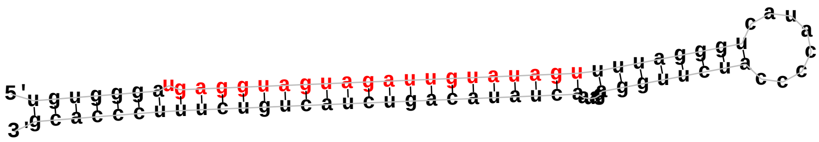

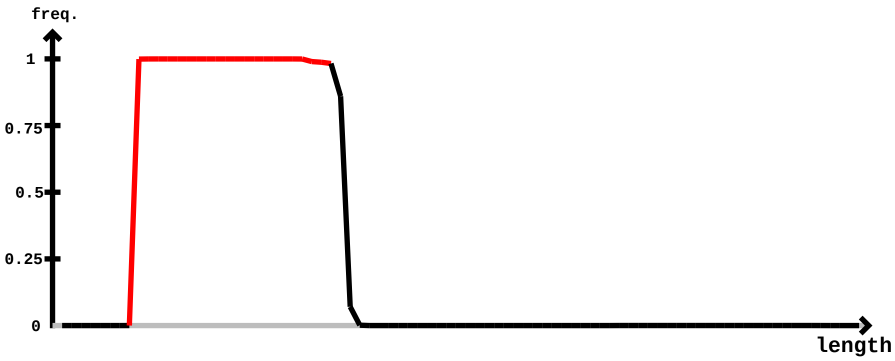


```
        oar-let-7f
5'- uguggga ugagguaguagauuguauagu uuuagggucauaccccaucuuggagauaacuauacagucuacugucuuucccacg   -3'    exp
    .((((((.(((((((((((((((((((((((((((((.......))))))))....))))))))))))))))))))))))))).    reads    mm    sample
    ......augagguaguagauuguauagu........................................................    10       0     seq
    ......Gugagguaguagauuguauaguu.......................................................    2        1     seq
    ......augagguaguagauuguauaguu.......................................................    33       0     seq
    .......ugagAuaguagauugua............................................................    1        1     seq
    .......ugagguGguagauugua............................................................    2        1     seq
    .......ugagguaguagauuguU............................................................    3        1     seq
    .......ugagguaguagauugua............................................................    216      0     seq
    .......ugagguaguagauugAau...........................................................    3        1     seq
    .......ugaAguaguagauuguau...........................................................    55       1     seq
    .......ugagguaguagauuguUu...........................................................    8        1     seq
    .......ugagguaguagauuguau...........................................................    6628     0     seq
    .......ugagguaguUgauuguau...........................................................    2        1     seq
    .......ugagguaguagauuguaua..........................................................    1752     0     seq
    .......ugagguaguagauugAauag.........................................................    1        1     seq
    .......ugagguaguagauuguauag.........................................................    3274     0     seq
    .......ugagguaguagauugAauagu........................................................    11       1     seq
    .......ugagguaguagauuguauagu........................................................    85681    0     seq
    .......ugagguaguagauuguauaguu.......................................................    550794   0     seq
    .......ugagguaguagauuguauaguuG......................................................    1834     1     seq
    .......ugagguaguagauuguauaguuu......................................................    46444    0     seq
    .......ugagguaguagauuguauaguuGu.....................................................    193      1     seq
    .......ugagguaguagauuguauaguuuu.....................................................    675      0     seq
    .......ugagguaguagauuguauaguuuua....................................................    11       0     seq
    ........gagguaguagauuguau...........................................................    4        0     seq
    ........gagguaguagauuguaua..........................................................    1        0     seq
    ........gagguaguagauuguauag.........................................................    1        0     seq
    ........gagguaguagauuguauagu........................................................    41       0     seq
    ........gagguaguagauuguauaguu.......................................................    278      0     seq
    ........gagguaguagauuguauaguuu......................................................    21       0     seq
    ........gagguaguagauuguauaguuG......................................................    2        1     seq
    ........gagguaguagauuguauaguuuu.....................................................    2        0     seq
    .........agguaguagauuguauag.........................................................    1        0     seq
    .........agguaguagauuguauagu........................................................    2        0     seq
    .........agguaguagauuguauaguu.......................................................    4        0     seq
    .........agguaguagauuguauaguuu......................................................    1        0     seq
```

oar-let-7f

```
ugugggaugagguaguagauuguauaguuuuagggucauaccccaucuuggagauaacuauacagucuacugucuuucccacg
.........agguaguagauuguauaguuG....................................................    8          1          seq
.........agguaguagauuguauaguuuu...................................................    1          0          seq
.........agguaguagauuguauaguuGu...................................................    63         1          seq
..........gguaguagauuguauaguu.....................................................    1          0          seq
.............................uuagggucauaccccaucuugga...............................    2          0          seq
........................................................cuauacagucuacugucuuu......    5          0          seq
........................................................cuauacagucuacugucuuuc.....    15         0          seq
........................................................cuauacagucuacugucuuucc....    102        0          seq
.........................................................uauacagucuacugucuuuc.....    1          0          seq
```
